# Isolation and identification of exosomes from feline plasma, urine and adipose-derived mesenchymal stem cells

**DOI:** 10.1186/s12917-021-02960-4

**Published:** 2021-08-12

**Authors:** Dongsheng Li, Huina Luo, Huimin Ruan, Zhisheng Chen, Shengfeng Chen, Bingyun Wang, Yong Xie

**Affiliations:** 1 VetCell Biotechnology Company Limited, Foshan, 528225 China; 2 School of Life Science and Engineering, Foshan University, Foshan, 528225 China; 3 Department of Obstetrics and Gynecology, The First People’s Hospital of Foshan, Foshan, 528000 China

**Keywords:** Feline, Exosomes, Mesenchymal stem cells, Plasma, Urine, Nanoparticle

## Abstract

**Background:**

Exosomes, internal proteins, lipids, and nucleic acids coated by phospholipid bilayer membranes, are one type of small extracellular vesicles, which can mediate cell-cell communication. In recent years, exosomes have gained considerable scientific interest due to their widely applied prospect in the diagnosis and therapeutics of human and animal diseases. In this study, we describe for the first time a feasible method designed to isolate and characterize exosomes from feline plasma, urine and adipose-derived mesenchymal stem cells.

**Results:**

Exosomes from feline plasma, urine and adipose-derived mesenchymal stem cells were successfully isolated by differential centrifugation. Quantification and sizing of exosomes were assessed by transmission electron microscopy, flow nano analysis and western blotting. Detected particles showed the normal size (30–100 nm) and morphology described for exosomes, as well as presence of the transmembrane protein (TSG101, CD9, CD63, and CD81) known as exosomal marker.

**Conclusions:**

The results suggest that differential centrifugation is a feasible method for isolation of exosomes from different types of feline samples. Moreover, these exosomes can be used to further diagnosis and therapeutics in veterinary pre-clinical and clinical studies.

**Supplementary Information:**

The online version contains supplementary material available at 10.1186/s12917-021-02960-4.

## Background

Exosomes, extracellular vesicles with a diameter of 30-120 nm, widely present in body fluids such as blood [1], saliva [2], cerebrospinal fluid [3], urine [4], milk [5], semen [6] and synovial fluid [7]. Exosomes deliver rich nucleic acid, protein and lipid content and participat in cell-to-cell communication [8, 9]. Exosomes are increasingly considered as biomarkers and prognostic factors of diseases, and have important clinical significance of diagnostic and therapeutic [10].

Exosomes isolated from biological fluids such as plasma and urine hold diagnostic potential [11]. Plasma-derived exosomes (Plasma-exo), which are simple collected, have no adverse effects on health, are considered diagnostic markers for several diseases such as oncology [12], hematonosis [13], angiocardiopathy [14] or ischemic disease [15]. The study of their content (protein or nucleic acid) components is also helpful for the treatment of diseases. Urine-derived exosomes (Urine-exo) are secreted by various cells in the urinary system and released into the urine. The changes of urinary exosome-derived miRNAs and proteins can be used as biomarkers in kidney diseases for monitoring the changes of diseases and judging prognosis, and also have important value in the disease treatment [16, 17].

Mesenchymal stem cells (MSCs) have emerged as a promising therapeutic strategy for several diseases. There is accumulating evidence suggesting their therapeutic effects are largely mediated by paracrine factors including cytokines, growth factors, and exosomes [18, 19]. Numerous studies have revealed that MSC-derived exosomes (MSC-exo) might represent a novel cell-free therapy with compelling advantages over MSCs such as lower immunogenicity and no tumorigenicity [20–23].

**Fig. 1:**
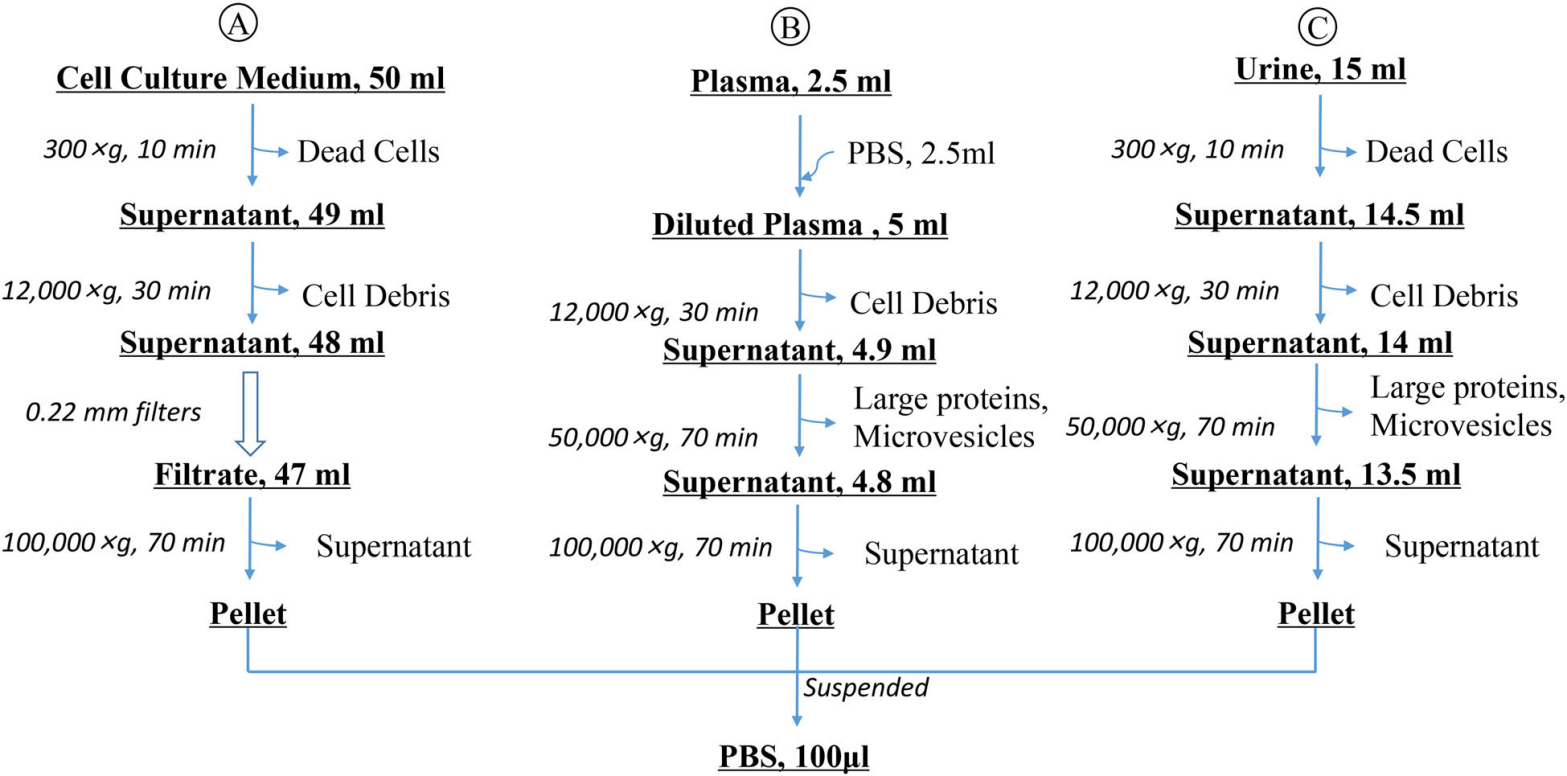
Procedures of the methods used for the isolation of exosomes from feline samples by differential ultracentrifugation. **A** The schema of the isolation procedure for feline adipose-derived mesenchymal stem cell-derived exosomes, **B** the schema of the isolation procedure for feline plasma-derived exosomes, and **C** the schema of the isolation procedure for feline urine-derived exosomes

In recent years, natural or artificially engineered exosomes as new carriers for drug delivery in clinics, have a good development prospect. It is particularly important to establish a fast, simple and stable separation method for the research and application of exosomes [24, 25]. The isolation and identification methods of different tissues-derived exosomes from dogs [26], horses [27] and cattle [28] samples have been established, but the exosomes from feline samples have been rarely reported. The objective of this study was to develop an efficient and robust method for MSC-exo, Plasma-exo and Urine-exo from feline samples (Fig. 1). This study provides comprehensive techniques such as transmission electron microscopy, flow nano analysis and western blotting to identify and characterize exosomes, allowing them to be quantified and sized, as well as characterized through specific morphology and a distinct protein expression.

## Results

### Identification of adipose-derived mesenchymal stem cells (AD-MSCs)

#### Differentiation of AD-MSCs

**Fig. 2:**
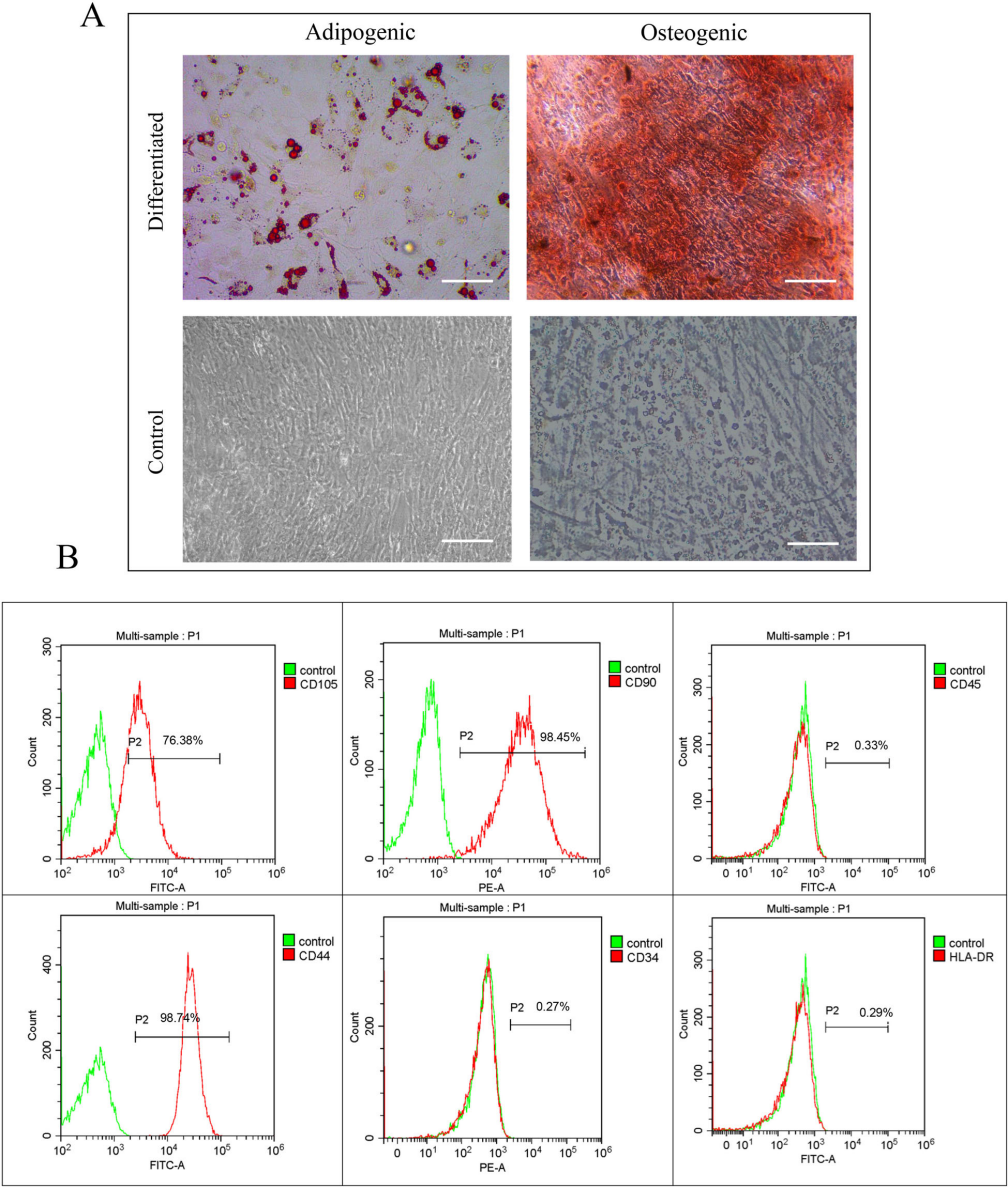
Identification of feline adipose-derived mesenchymal stem cells. **A** Adipogenic and osteogenic differentiation of feline AD-MSCs. AD-MSCs were positive for Oil red-O staining and alizarin red staining. Scale bars, 50 μm. **B** Surface markers of feline AD-MSCs. Based on flow cytometric analysis, surface molecule markers CD44, CD90, and CD105 were highly expressed on feline AD-MSCs, whereas the expression of hematopoietic stem cell markers CD34, leukocyte common antigen CD45, and major histocompatibility complex HLA-DR were rarely expressed

After induction with adipogenic medium for 14 days, AD-MSCs gradually changed from fibroblast-like cells to flattened cells, and many different sizes lipid droplets appeared in the cytoplasm. Cellular staining was positive and the multiple lipid droplets in differentiated cells were stained red by staining with Oil red-O. After incubation with osteogenic medium for 5 days, MSCs exhibited obvious morphological alterations. Calcium nodules appeared on the 10th day of induced differentiation and tightly packed colonies forming nodule-like structures were observed and deposition of calcium in these cells was observed by staining with alizarin red (Fig. 2A).

#### Flow cytometry analysis of AD-MSCs

*AD-MSCs* were highly-expressed mesenchymal stem cell surface markers CD44, CD90 and CD105, while for the lowly-expressed haematopoietic stem cells surface markers CD34, leukocyte common antigen CD45 and major histocompatibility complex class II HLA-DR (Fig. 2B). That is, the isolated and cultured cells conformed the characteristics and identification criteria of mesenchymal stem cells.

### Transmission electron microscopy (TEM)

**Fig. 3:**
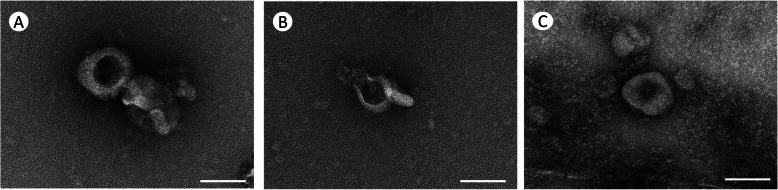
Transmission electron microscopy of exosomes from feline samples. A representative TEM image of isolated exosomes from feline adipose-derived mesenchymal stem cell culture medium(**A**), from feline plasma (**B**), and from feline urine (**C**). Exosomes isolated by differential ultracentrifugation were cup-shaped and in size from 30 to 100 nm

TEM confirmed 3 different soures-derived exosomes showed the cup-shaped spherical morphology with of exosomal vesicles that are concave in the middle (Fig. 3). The vesicles observed ranged in size from 30 to 100 nm.

### Flow nano analyzer

**Fig. 4:**
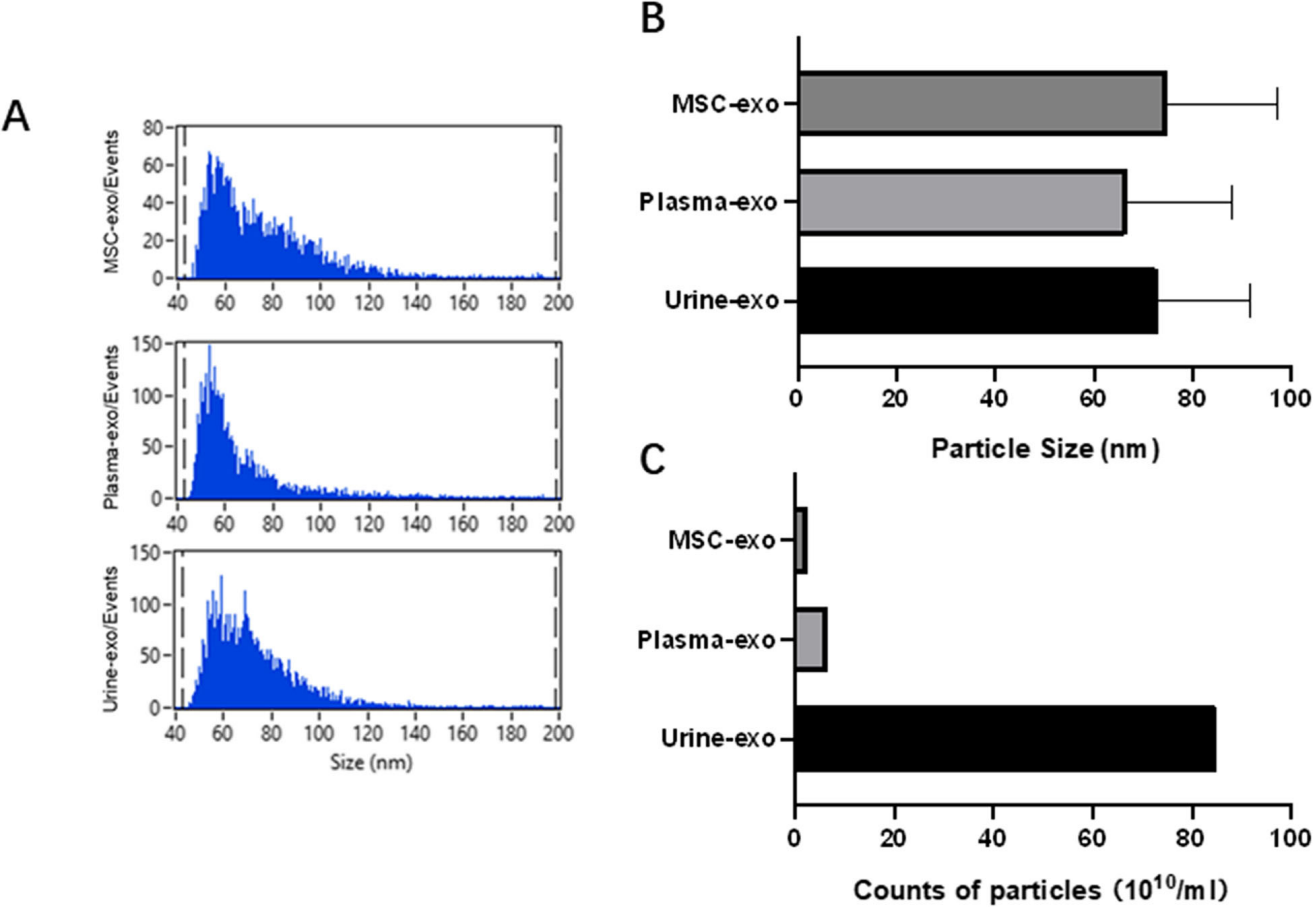
The size and concentration of feline samples-derived exosomes measured by flow nano analyzer. **A** Nano track analysis size distribution of exosomes isolated from feline adipose-derived mesenchymal stem cell culture medium, feline plasma, and feline urine. **B** Diameter of isolated particles (exosomes). The mean diameters of exosomes from feline adipose-derived mesenchymal stem cell culture medium, plasma, and urine were 74.76 nm, 66.62 nm, and 72.88 nm, respectively. **C** Counts of particles (exosomes). The concentration of exosomes from feline adipose-derived mesenchymal stem cell culture medium, plasma, and urine were 2.62 × 10^10^ /ml, 6.42 × 10^10^ /ml, and 8.49 × 10^11^ /ml, respectively

The exosomes from MSCs cell culture medium, plasma, and urine exhibited an ideal mean diameter of 74.76 nm, 66.62 nm, and 72.88 nm, a concentration of 2.62 × 10^10^ /ml, 6.42 × 10^10^ /ml, and 8.49 × 10^11^ /ml, as detected by Flow NanoAnalyzer (Fig. 4).

### Western blotting

**Fig. 5:**
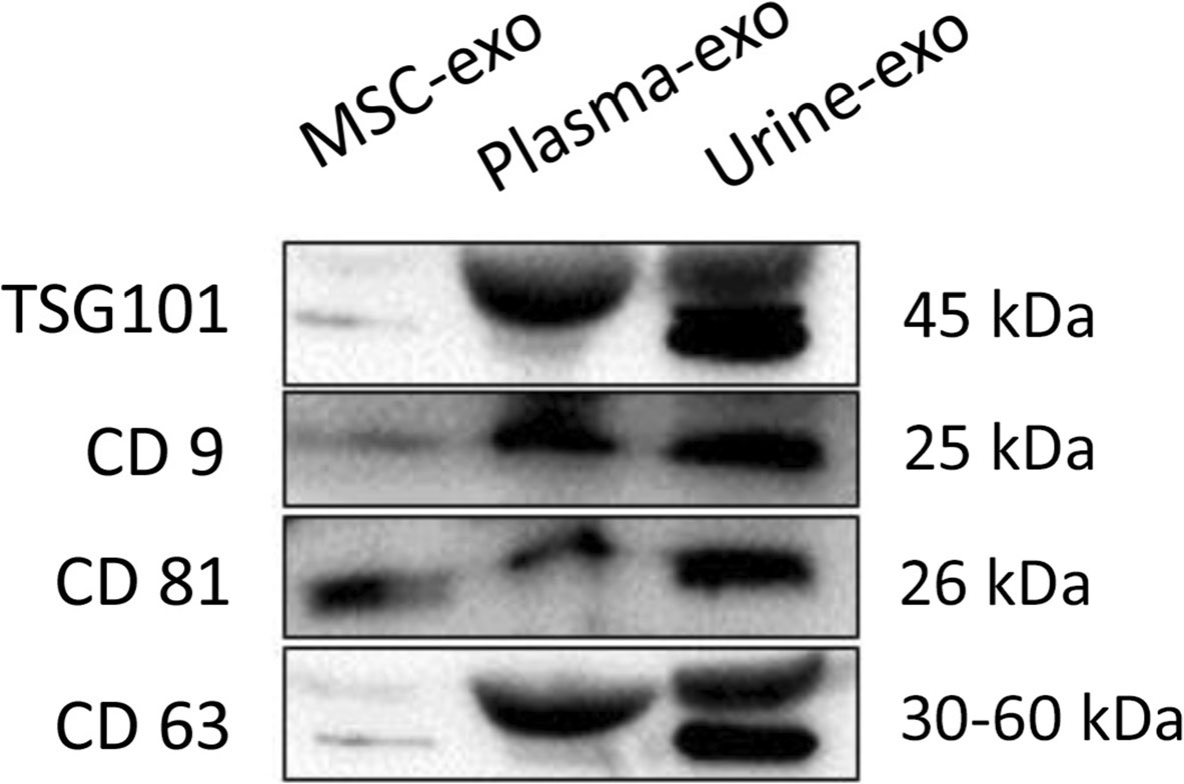
Western blotting analysis of exosomes from feline samples with anti-TSG101, anti-CD9, anti-CD63, and anti-CD81. Surface Markers TSG101, CD9, CD63, and CD81 positively expressed in exosomes secreted from feline adipose-derived mesenchymal stem cell culture medium, plasma, and urine

Our analysis revealed detection of four surface-marker proteins (TSG101, CD9, CD63, and CD81), with results showing all samples isolated by our ultrafiltration technique were positive for TSG101, CD9, CD63, and CD81, indicating the presence of exosomal marker proteins (Fig. 5 and Additional file 1).

## Discussion

Exosomes are released by virtually every cell type in the body cells into biological fluids in vivo and cell culture conditioned media in vitro [29, 30]. Exosomes have been shown to be key mediators of cell to cell communication, delivering a distinct cargo of lipids, proteins and nucleic acids that reflects their cell of origin [31, 32]. As a new biomarker, exosomes have been widely used in the diagnosis and therapeutics of human diseases, but there are few researches in related fields of pet medical. The research interest in exosomes is continuously increasing however the lack of standard methods for isolation and quantification, limits the reliability and reproducibility of exosome use [33, 34].

This study provided a method based on differential centrifugation of exosome isolation for 3 different biofluids from feline samples, laying a foundation for the application of exosomes in disease diagnosis and treatment of pet cats in the future. The differential centrifugation method is based on the difference in size and density between the exosome sample and other substances, through a series of centrifuges with different centrifugal forces and different centrifugal time lengths, non-exosomes are gradually removed after precipitation, and then exosomes are precipitate by ultracentrifugation and re-suspended finally [35, 36]. Ultracentrifugation is the most widely used method for exosome isolation and was once called the “gold standard” for exosome preparation [37, 38]. Due to its simple operation and stable separation effect, about more than half of exosomes related researcher used this method to extract exosomes [39].

In this study, the ultrastructure, particle size and surface markers of exosomes were identified by transmission electron microscopy, flow nano analyzer and western blot. The results showed that the three exosomes were round or elliptic vesicles with membranous structures around the vesicles, similar in shape to those previously described in mammals. The particle size of Urine-exo detected by flow nano analyzer is the largest of the three exosome samples, while Plasma-exo is the smallest, but all within the range of 30–100 nm. Compared to plasma and urine samples, the number of exosomes found in MSCs cell culture medium was significantly lower. This may be because the volume of 50 ml cell culture medium is too small, and a larger volume of medium is needed to obtain higher production of exosomes. Tetraspanins (including CD81, CD63 and CD9 protein) are common exosomal specific markers for extracellular vesicles such as exosomes and were suggested by the International Society of Extracellular Vesicles (ISEV) for the identification of exosomes [27]. As a cytosolic protein, Tumor Susceptibility Gene 101 (Tsg101) is involved in multivesicular body formation of exosome, is considered to be another important exosome marker [40]. Our western blotting result showed that the marker proteins were detected to be all positive in exosomes from 3 different biofluids. But all proteins signal strengths of MSC-exo are weaker than those in the serum and urine, probably because number of exosomes are fewer of them. Therefore, combined with the above results, it is demonstrated that the methods of exosome isolation we established is feasible and effective, allowing nanoparticles to be analysed in downstream applications.

## Conclusions

Overall, our results evidence the feasibility to easily isolate exosome from the supernatants of feline adipose derived mesenchymal stem cells, as well as from plasma and urine of feline. This method for isolating exosomes from feline samples can be used to further diagnosis and therapeutics in veterinary pre-clinical and clinical studies.

## Methods

### Isolation, culture and identification of adipose-derived mesenchymal stem cells

Abdominal subcutaneous adipose tissues were collected aseptically at Affiliated Animal Hospital, Department of Veterinary Medicine of Foshan University. The tissues were cut into tissue blocks about 1 mm^2^ in size and were digested with 1 mg/mL collagenase type I at 37 °C for 2 ~ 3 h. The digestive juices were filtered with 200-mesh cell strain and centrifuged at 800×g for 5 min to collect AD-MSCs. Approximately 5000 isolated suspended cells per cm^2^ were transferred to cell culture flask (Corning, USA) in Dulbecco’s Modified Eagle’s Medium supplemented with 10% exosome-free Fetal Bovine Serum (FBS, Biological Industries, Israel), 1% Pen-Strep (Gibco, USA), and 1% L-glutamine (Gibco, USA) and placed into the incubator at 37 °C in a humidified incubator containing 5% CO_2_. After 24 h, the medium was replaced for the first time to remove most of the blood cells and replaced every 3 d thereafter. AD-MSCs were digested with 0.25% trypsin and passaged routinely when 80 ~ 90% confluence was reached.

The AD-MSCs were characterized by multipotential differentiation and flow cytometry analysis. In vitro adipogenic and osteogenic differentiation were examined using MSCs Adipogenic Differentiation Kit (Cyanogen, China) and MSCs Osteogenic Differentiation Kit (Cyanogen, China) following the manufacturer’s protocol for each kit. Cells were stained with Oil Red O solution to assess adipogenic differentiation and alizarin red solution to assess osteogenic differentiation.

Flow cytometry analysis was performed using a CytoFLEX flow cytometry instrument (Beckman, USA). Data acquisition and analysis was performed with CytExpert (Beckman, USA). Briefly, AD-MSCs of passage 2 were stained for 30 min with FITC -conjugated or phycoerythrin (PE)-conjugated monoclonal antibodies at 37 °C. The following monoclonal antibodies were used: anti-CD34-PE (cat.no.ab23830; Abcam), anti-CD44- FITC (cat.no.MA1–10229; Invitrogen), anti-CD45-FITC (cat.no.ab27287; Abcam), anti-CD90-PE (cat.no.11–0900-81; Invitrogen), anti-CD105-FITC (cat.no.ab11415; Abcam),and anti-HLA-DR-FITC (cat.no. L243–347400; BD Biosciences). Chilled PBS was used to wash and remove unbound antibodies, and then a total of 2 × 10^5^ cells from each sample tube were acquired for analysis using Flow Cytometer.

### Preparation of cell culture medium samples

FBS added to the cell culture medium should be depleted of exosomes by ultracentrifugation at 120000 x g over night at 4 °C prior to use.50–80% confluent AD-MSCs at passage 2–5 were washed twice in PBS and further cultured in an exosome-free medium as described above. Briefly, cell culture medium was harvested after 48 h of incubation with exosome- free medium and stored at − 80 °C for subsequent experiments.

### Preparation of plasma samples

Samples were mixed from 3 female and 2 male felines presented at Affiliated Animal Hospital, Department of Veterinary Medicine of Foshan University. Blood samples are collected into acollections tubes containing anticoagulant and the cell components were removed by centrifugation (800×g, 4 °C, 15 min). The supernatant was diluted with phosphate buffered saline of the same volume (1:1) and stored at − 80 °C for subsequent experiments.

### Preparation of urine samples

Urine samples were mixed from 1 female and 2 male felines presented at Affiliated Animal Hospital, Department of Veterinary Medicine of Foshan University. Samples are collected into tubes and stored at − 80 °C for subsequent experiments.

### Isolation of exosomes

Exosomes were isolated by differential centrifugation. Briefly, Cell culture medium (50 mL) were centrifuged at 4 °C, 300×g for 10 min to remove dead cells, followed by centrifuging at 12,000×g for 30 min at 4 °C to remove cell debris. Supernatant was collected and filtered through 0.22 mm filters (Merck Millipore, USA) to remove contaminating microvesicles larger than 200 nm. Following this, the filtered supernatant was transferred to new polycarbonate tubes for ultracentrifugation in ultra-speed centrifuge (Beckman Coulter XL-90, SW28Ti rotor; Beckman Coulter; USA) at 100,000×g for 70 min at 4 °C and if not completely full add PBS. Discard the supernatant. For maximal exosome retrieval, resuspend the exosome enriched pellet repeatedly in 100 μl PBS.

Diluted plasma samples (5 ml) were centrifuged at 12,000×g for 30 min at 4 °C to remove cell debris. Transfer the supernatant to new ultracentrifuge tubes and if not completely full add PBS. Clarified supernatant was ultracentrifuged at 50,000×g for 70 min at 4 °C to remove large proteins and microvesicles. Following this, supernatant was ultracentrifuged at 100,000×g for 70 min at 4 °C. Discard the supernatant. For maximal exosome retrieval, resuspend the exosome enriched pellet repeatedly in 100 μl PBS.

Urine samples (15 ml) were centrifuged at 4 °C, 300×g for 10 min to remove dead cells, followed by centrifuging at 12,000×g for 30 min at 4 °C to remove cell debris. Transfer the supernatant to new ultracentrifuge tubes and if not completely full add PBS. Clarified supernatant was ultracentrifuged at 50,000×g for 70 min at 4 °C in ultra-speed centrifuge remove large proteins and microvesicles. Following this, supernatant was ultracentrifuged at 100,000×g for 70 min at 4 °C. Discard the supernatant. For maximal exosome retrieval, resuspend the exosome enriched pellet repeatedly in 100 μl PBS.

### Transmission electron microscopy (TEM)

Exosome samples were diluted in PBS, dropped on a carbon-coated copper grid, and then stained with 1% uranyl acetate for 1 min. Grids were imaged under a Hitachi H-7650 transmission electron microscope.

### Flow nano analyzer

Exosome samples were diluted 1:100 and analyzed using the Flow Nano Analyzer (NanoFCM Inc.) according to manufacturer’s protocol. Briefy, the lasers were calibrated using 200 nm control beads (NanoFCM Inc.), which were then analyzed as a reference for particle concentration. Additionally, a mixture of diferent sized beads (NanoFCM Inc.) was analyzed to set reference for size distribution.

### Western blotting

Exosome samples were denatured in protein loading buffer (10% sodium dodecyl sulfate (SDS), 250 mM Tris-HCl (pH 6.8), 0.5% Bromophenol blue, 50% glycerin, 5% β-Mercaptoethanol) at 95 °C for 10 min. Proteins were separated by 10% sodium dodecyl polyacrylamide gel electrophoresis (SDS-PAGE), and were then transferred to polyvinylidene fluoride (PVDF) membranes (Merck Millipore, USA). The membranes were blocked with 5% non-fat milk in Tris-buffered saline containing 0.1% Tween-20 for 1 h at room temperature and afterwards incubated at room temperature for 1 h with antibodies against TSG101 (Santa Cruz, sc-7964, 1:1000), CD81 (Affinity, DF2306, 1:1000), CD63 (Santa Cruz, sc-5275, 1:1000) and CD9 (Affinity, AF5139, 1:1000), followed by incubation with horseradish peroxidase conjugated secondary antibodies at room temperature for 1 h. Luminescent visualizationwas done using an ECL kit (Tanon, China) to identify immunoreactive protein bands.

## Supplementary Information

**Additional file 1.** Western blot instructions and original images.

## Data Availability

The datasets used and/or analysed during the current study are available from the corresponding author upon reasonable request.

## Abbreviations

AD-MSCs: Adipose-derived mesenchymal stem cells
FBS: Fetal bovine serum
ISEV: International society of extracellular vesicles
MSCs: Mesenchymal stem cells
MSC-exo: Mesenchymal stem cells derived exosomes
Plasma-exo: Plasma derived exosomes
PVDF: Polyvinylidene fluoride
SDS: Sodium dodecyl sulfate
Tsg101: Tumor susceptibility gene 101
Urine-exo: Urine derived exosomes
