# Supplementary material for: Isolation and identification of exosomes from feline plasma, urine and adipose-derived mesenchymal stem cells
Source: BMC Vet Res. 2021 Aug 12;17:272. doi: 10.1186/s12917-021-02960-4 (PMC8359027; doi:10.1186/s12917-021-02960-4)
Supplement: Supplementary file 1 — Additional file 1. Western blot instructions and original images. [file 12917_2021_2960_MOESM1_ESM.pptx]

## Slide 1
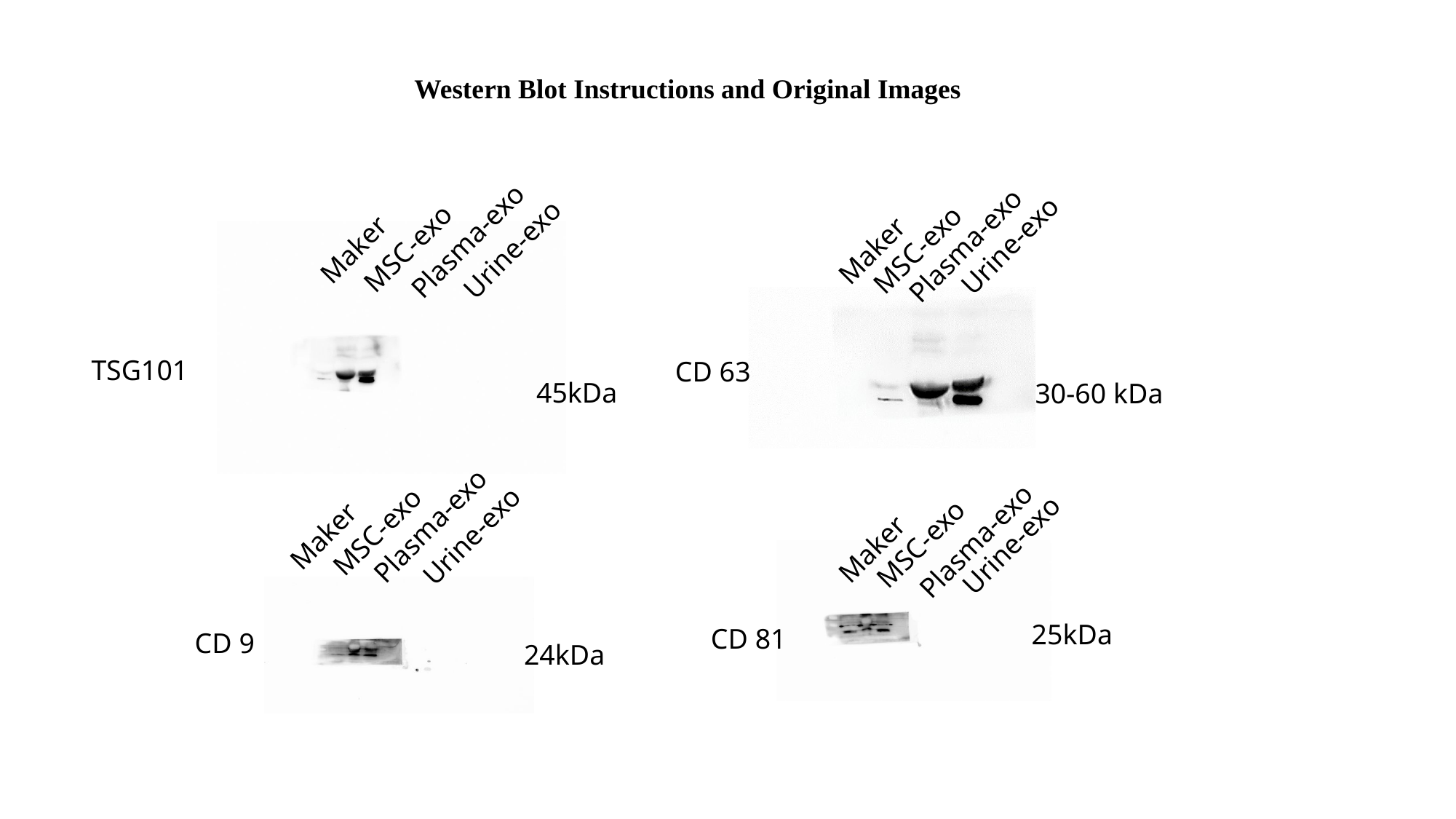

Western Blot Instructions and Original Images
Plasma-exo
Plasma-exo
Urine-exo
MSC-exo
Urine-exo
MSC-exo
Maker
Maker
TSG101
CD 63
45kDa
30-60 kDa
Plasma-exo
MSC-exo
Maker
Urine-exo
Plasma-exo
MSC-exo
Urine-exo
Maker
25kDa
CD 81
CD 9
24kDa
